# Cherry Tomato Production in Intelligent Greenhouses—Sensors and AI for Control of Climate, Irrigation, Crop Yield, and Quality

**DOI:** 10.3390/s20226430

**Published:** 2020-11-11

**Authors:** Silke Hemming, Feije de Zwart, Anne Elings, Anna Petropoulou, Isabella Righini

**Affiliations:** Business Unit Greenhouse Horticulture, Wageningen University & Research (WUR), 6708PB Wageningen, The Netherlands; feije.dezwart@wur.nl (F.d.Z.); anne.elings@wur.nl (A.E.); anna.petropoulou@wur.nl (A.P.); isabella.righini@wur.nl (I.R.)

**Keywords:** artificial intelligence, sensors, resource use efficiency, tomato yield, indoor farming, autonomous greenhouses, climate control, irrigation control, remote control, data driven growing

## Abstract

Greenhouses and indoor farming systems play an important role in providing fresh and nutritious food for the growing global population. Farms are becoming larger and greenhouse growers need to make complex decisions to maximize production and minimize resource use while meeting market requirements. However, highly skilled labor is increasingly lacking in the greenhouse sector. Moreover, extreme events such as the COVID-19 pandemic, can make farms temporarily less accessible. This highlights the need for more autonomous and remote-control strategies for greenhouse production. This paper describes and analyzes the results of the second “Autonomous Greenhouse Challenge”. In this challenge, an experiment was conducted in six high-tech greenhouse compartments during a period of six months of cherry tomato growing. The primary goal of the greenhouse operation was to maximize net profit, by controlling the greenhouse climate and crop with AI techniques. Five international teams with backgrounds in AI and horticulture were challenged in a competition to operate their own compartment remotely. They developed intelligent algorithms and use sensor data to determine climate setpoints and crop management strategy. All AI supported teams outperformed a human-operated greenhouse that served as reference. From the results obtained by the teams and from the analysis of the different climate-crop strategies, it was possible to detect challenges and opportunities for the future implementation of remote-control systems in greenhouse production.

## 1. Introduction

Greenhouses and indoor farming systems play an important role in providing fresh food, such as fruits and vegetables being high in vitamins and minerals. Greenhouses combine high crop production per unit area with a high water use efficiency per unit of produce [1], but at the cost of high energy demand [2] and high investments. Greenhouse production is increasing in many countries worldwide [3] to provide fresh food, preferably produced locally [4]. However, educated and experienced labour is scarce. In many countries skilled labour to oversee all aspects of greenhouse crop production [5] is lacking. The current COVID-19 pandemic has shown that the availability of seasonal labor is critical for horticulture production [6]. The need of highly educated and experienced crop managers has increased, just like the need for more automation and remote control of greenhouses and other farming systems. As farms become larger, or temporarily less accessible, remote monitoring of climate, irrigation, and crop status becomes more important. More sensors and objective digital information become crucial for crop managers to take informed decisions to reach high crop yield with high quality. Due to climate change, natural resources such as water and (fossil-based) energy are becoming scarcer and improving the resource efficiency becomes urgent. A greenhouse grower needs to make many decisions to simultaneously maximize production and to minimize resources. Crop and greenhouse climate models and/or new intelligent algorithms can help the grower to oversee all information available and to support complex decisions to predict yields and resource use.

Today’s high-tech greenhouses are equipped with different standard sensors for monitoring light, temperature, humidity, and CO_2_ and for actively controlling different actuators (e.g., lighting, screening, heating, ventilation, cooling, CO_2_ dosing, fogging, dehumidification, irrigation, and fertilizer dosing) in order to control all growth factors important for crop production at every moment. Today’s growers determine the climate, irrigation and crop management strategies based on experience and defines the setpoints for climate and irrigation control manually. Actuators then operate based on setpoints configured in a process computer, while sensors give feedback on measured data for the control loop. Additional sensors monitoring crop status are able to provide the grower with further information on photosynthesis rate [7], sap flow and hydraulic status [8] and leaf temperature [9] to be added to his manual decisions. Automated greenhouse climate control algorithms have already been developed in the past and are today widely introduced in modern high-tech greenhouses [10,11,12,13,14,15,16,17,18,19,20,21,22]; however, automated control on crop status are still in its infancy.

Dynamic climate models have been developed [10,18,23,24,25,26,27,28] which act as a digital twin of the real greenhouse. An overview of today’s greenhouse climate models is given in a previous study [29]. Mechanistic models give the opportunity to be used for intelligent decision support on climate control actions. Simulations of past or future scenarios provide information on how a different climate control in the past could have improved crop production and which actions are required to reach a certain crop production goal in the future. These models can also be coupled with intelligent algorithms to automatically determine climate setpoints, an action that is currently performed manually by the grower. In order to control crop production by an automated algorithm, mechanistic greenhouse climate and crop models can be used and coupled with a real greenhouse, to send automatically determined setpoints via a process computer to control the different actuators. Such optimum control experiments have been conducted with tomato [30,31], sweet pepper [32,33] or pot plants [34] in the past.

The crop has a central role in every greenhouse production system. Crop management decisions and actions are mostly taken by the greenhouse staff. Manual labor is still required for planting, crop training, leaf and fruit pruning, and fruit harvesting in greenhouses with high-wire vegetable production. While manual labor requirement is high, crop management decisions can be supported. Since experienced and well-trained crop managers are scarce, crop simulation models can play a role in decision making. An overview of greenhouse crop models and modelling approaches are given in other studies [35,36,37,38,39,40,41,42,43]. Crop models can be used as virtual representations of reality [44]. They can be used to simulate different growing conditions and crop management strategies and predict crop development and yield, and fruit quality. Crop models can help to understand the crop behavior under different growing conditions and can support the grower in making decisions.

AI algorithms are widely used in horticultural research and have recently been implemented in practice. Main application fields are plant stress detection [45], fruit detection or counting [46], pest, disease, or weed detection [47,48,49], yield prediction or harvesting [50,51]. Different camera and spectroscopy systems in different spectral ranges are used for detection, different computer vision and machine learning algorithms are used for analysis. However, the use of AI for greenhouse crop production control is still limited [52,53,54,55,56,57]. Recently a benchmark experiment has been conducted to use artificial intelligent (AI) algorithms to optimize net profit of a cucumber crop in a greenhouse experiment during the first Autonomous Greenhouse Challenge in 2018. In that experiment the winning AI algorithm outperformed the human decisions of experienced growers [58]. Crop production (class A: commercially sellable fruits) was increased by 6% and net profit by 17% compared to the growers who acted as a reference.

In the current paper the results of the second Autonomous Greenhouse Challenge conducted in six high-tech greenhouse compartments at Wageningen University & Research, Bleiswijk, in The Netherlands in 2020, was described. The challenge was designed to make further breakthroughs in fresh food production with fewer resources using AI algorithms for and automatic and remote control of a greenhouse crop production. While the first experiment [58] was simpler with only a 3–4 months control of cucumber production, this second experiment was more complex to prove the value of AI control over a longer six months period. A different crop had to be grown, cherry tomatoes require more complex control since it can be controlled not only on yield but also on product quality. While in the first experiment the crop was grown during a summer–autumn growing season, in this second experiment the crop was grown during winter–spring–summer. Other challenges were added such as no fixed product prices, but prices were dependent on fruit quality and fruit quality was dependent on nutrient control, nutrient control was an offtrade of yield (more income but lower prices) and product quality (high prices but lower yield). Comparable to the first experiment [58], five multi-disciplinary international teams automatically controlled their own greenhouse compartment remotely, a sixth team of local experienced human reference growers acted as manually controlled reference. The goal of the experiment was to maximize net profit by realizing high yield and product prices and minimize resource use and costs.

The aim of this paper is to describe the results of the experiment in terms of net profit, yields and resource use, to analyze different climate and crop management strategies, to explain the results with the help of a digital twin model of a virtual greenhouse, and to detect possible improvements of automatic control for the future. The experiment provided a valuable public dataset which can be used for future AI training purposes and which can be found at Supplementary Materials: https://doi.org/10.4121/uuid:88d22c60-21b3-4ea8-90db-20249a5be2a7.

## 2. Materials and Methods

### 2.1. Greenhouse Compartments and Equipment

**Figure 1:**
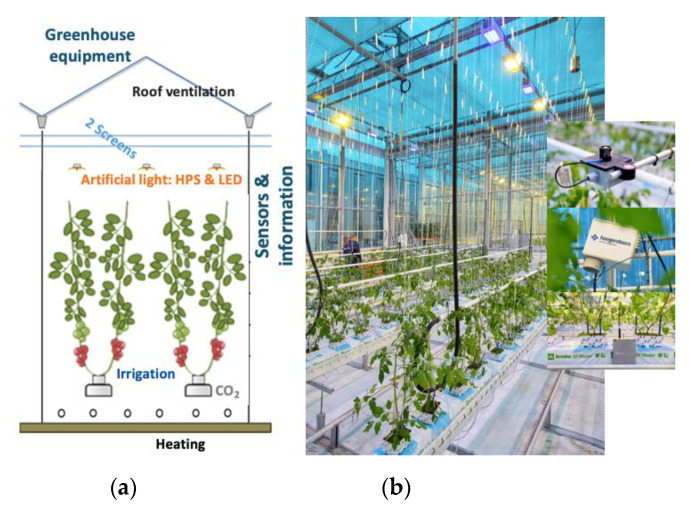
Greenhouse experimental compartments, 96 m^2^ floor area (76.8 m^2^ crop-growing area) provided with different equipment. (**a**) Scheme of compartment with crop and equipment: roof ventilation, two screens, artificial light (high-pressure-sodium (HPS), LED), irrigation system, CO_2_ supply, two heating systems. (**b**) Picture of one compartment with the young cherry tomato crop after the transplant with equipment and sensors. Principle of the set-up earlier described in [58].

The experiment described in this paper has been conducted in a high-tech greenhouse with six identical compartments at Wageningen University & Research, Bleiswijk, The Netherlands. Each compartment measured 96 m^2^ floor area and was equipped with technology comparable to commercial high-tech greenhouses (Figure 1). The set-up is comparable to our earlier experiment described in [58] however, differences in the lighting systems apply. The artificial lighting system consisted of 6 high-pressure-sodium (HPS) lamps (capacity of 100 μmol/m^2^/s, ePapillon fixture of Lights Interaction Agro with a Philips Master GreenPower Plus 1000W EL light bulb, The Netherlands) and 8 multi-spectrum controllable LED lamps (capacity from 0 to 109 μmol/m^2^/s, from which 12 μmol/m^2^/s is far-red and therefore not counted as PAR, the other spectrum channels are max. blue = 11, red = 49, and white = 37 μmol/m^2^/s; Elixia, Heliospectra, Sweden). Power supply to the LED lamps was coupled to the power supply of the HPS, meaning that LED lamps could only be used additionally to the HPS-lamps. For control of natural light and energy saving two types of inside moveable screens (LUXOUS 1547 D FR energy screen and OBSCURA 9950 FR W light blocking screen, Ludvig Svensson, Sweden) were present. For temperature control a rail pipe heating system on the floor and a pipe heating system at crop height (peak capacity 180 and 30 W/m^2^, respectively) were installed, both controllable independently. Next to that, a continuous roof ventilation (ventilation area of 0.3 m^2^ opening per m^2^ greenhouse), equipped with anti-thrips netting was available and a fogging system (maximum capacity of 330 g/m^2^/h), and CO_2_ supply (maximum capacity 15 g/m^2^/h) were mounted. Plants were grown in rockwool cubes, placed on rockwool slabs (Grodan GT Master, Grodan, The Netherlands), located on elevated gutters. Irrigation water, premixed with nutrients was supplied with drippers, pressurized by an on/off controlled irrigation pump. The surplus of irrigation water (drain) was collected in the gutter and measured in terms of quantity, EC, and pH.

### 2.2. Greenhouse Control

**Figure 2:**
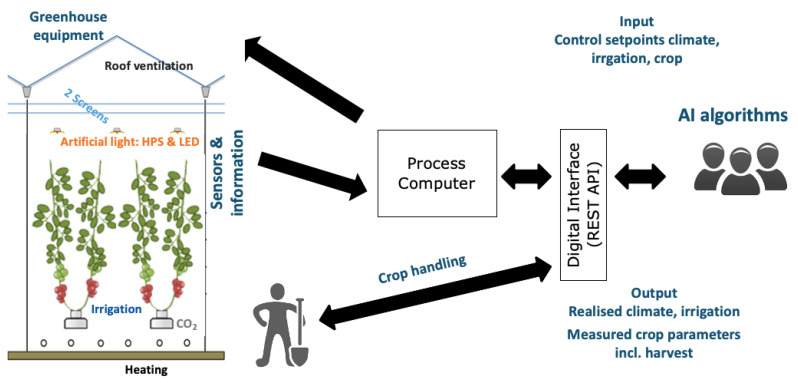
Scheme of data exchange from the teams and their AI algorithm via a digital interface (REST API) towards the process computer and the greenhouse actuators and data from sensors via the same way back, data exchange between teams and workers on crop handling, and measured crop parameters. Principle of the set-up earlier described in [58].

Five international teams (Automatoes, AiCU, DIGILOG, IUA.CAAS, The Automators) controlled their own greenhouse compartment (described here as compartment 306, 302, 305, 304, 301, respectively) remotely based on their own algorithms. A sixth greenhouse compartment (303) was manually controlled by Dutch growers and served as a reference. Competing teams used their own control algorithms to determine the climate and irrigation control setpoints. Setpoints teams could control were comparable to our earlier experiment described in [58]: artificial lighting HPS and LED (on/off; 0% or 100%) and if on, the lighting intensity of the four LED spectrum channels (blue, red, far-red, white) (0–100%), energy screen position (0–100%), blackout screen position (0–100%), minimum rail pipe temperature (°C), minimum crop pipe temperature (°C), minimum ventilation opening (%), ventilation temperature (°C), humidity deficit setpoint (g/m^3^), CO_2_ concentration (ppm), and time between subsequent irrigation turns (min). Setpoints were sent via a digital interface (LetsGrow.com, The Netherlands) to a process computer (IISI, Hoogendoorn, The Netherlands), which then operated the equipment accordingly (Figure 2). A nutrient solution was prepared by a central fertigation computer and sent to a daily storage tank per compartment before being provided to the crop with drippers. Based on GroSense sensor data (Grodan, The Netherlands) obtained in the rockwool slabs, and detailed chemical analysis of the drain water, provided every fortnight, the teams could send requests to change the composition, EC, and pH of the nutrient solution. Different sensors in the greenhouse collected data on climate and irrigation automatically (see Section 2.3) and returned them to the teams via the process computer and the digital interface (Representational State Transfer Application Programming Interface REST API). Staff in the greenhouse collected data on crop parameters manually and entered these observations on a tablet (see Section 2.4). This information was sent via the digital interface as well. Based on these observations the teams generated crop management settings that were passed also by the digital interface. On a weekly basis, these settings were translated to crop management instructions for the humans in the greenhouse.

The competing teams developed hybrid systems and combined expert policies with control and predictive algorithms to support their growing strategies. Diverse algorithms were explored and applied, varying from conditional, rule-based algorithms, to data enabled predictive control (DeePC), long short-term memory networks (LSTM), bidirectional LSTM, reinforcement learning, and imitation learning.

### 2.3. Sensors

In each greenhouse compartment standard sensors continuously measured data. Standard sensors were comparable to our earlier experiment described in [58] and can be divided into:

1. Sensors monitoring outside weather parameters: cumulative outside global radiation (J/cm^2^/d), outside photosynthetically active radiation PAR (μmol/m^2^/s), air temperature outside (°C), outside relative humidity (%), and wind speed (m/s);
2. Outside weather forecast parameters: outside global radiation forecast (W/m^2^), outside air temperature forecast (°C), outside relative humidity forecast (%), and wind speed forecast (m/s);
3. Sensors monitoring inside climate parameters and equipment status: lamp status (on/off) of both lighting systems (HPS and LED) and intensity of the four channels of LED lighting (0–100%), energy and black-out screen position (%), air temperature inside (°C), heating pipe temperature (°C), heating power used (W/m^2^) for both heating systems, air absolute humidity inside (g/m^3^), CO_2_ dosage (kg/ha/h);
4. Sensors monitoring fertigation parameters and equipment status: irrigation supply amount (L/m^2^), drain amount (L/m^2^), drain EC (dS/m), and drain pH (−), EC in slab (dS/m), pH in slab (-), and temperature in slab (°C).

Both setpoints for control of equipment and measured data were exchanged at a 5-min-interval. In addition, the following daily data was calculated from the measured data: inside PAR sum (mol/m^2^), heating energy used (kWh/m^2^), electricity used (kWh/m^2^), CO_2_ dosage (kg/m^2^), water consumption (L/m^2^). Measurements and calculations were sent back to the teams via a digital interface (Figure 2).

Teams could install additional sensors at the start of the experiment. They chose different types of sensors, such as additional aspirated measurement boxes for indoor temperature, humidity and CO_2_, indoor PAR meters, crop temperature, pyranometers, slab weight sensors, additional substrate water content, EC and temperature sensors, stem diameter, sap flow meters, crop weight, infrared leaf thermometer, plant temperature camera’s, RGB camera’s, and thermal imaging camera’s (pictures only, video streaming was not allowed). Data from additional sensors was received by teams at different time intervals depending on the parameters and devices and acquired via the specific supplier companies’ interfaces and/or arranged by teams via separate interface.

### 2.4. Crop

The experiment was conducted with an indetermined type of cherry tomato crop. Seedlings cv. “Axiany” (Axia Seeds, The Netherlands) were sown on 19 October 2019, grafted on Maxifort rootstock, planted in rockwool cubes and were transplanted to the greenhouse compartments on 16 December 2019. Teams took over remote control on 20 December 2019. The crop was grown in a high-wire growing system. Initial plant density and stem density were determined by the teams in advance and varied between 2.6 and 4.0 stems/m^2^, all teams opted for a 2-stem young plant. The reference started with 4.0 stems/m^2^. Changes in stem density during the cropping period were different for the teams in time. The first harvest was on 13 February 2020, and the last harvest was for all teams set to 29 May 2020. Based on this last harvest date, the date of topping (removal of head of the crop) had to be chosen by the teams and differed from 16 to 30 April 2020. The crop in the reference compartment was topped on 16 April 2020.

Teams sent weekly instructions with regards to stem density, and fruit and leaf pruning in the top of the canopy to the greenhouse staff. Stem density over time ranged from 2.6 to 8.0 stems/m^2^. Fruit pruning strategies led to a different maximum plant load of 500 to 800 fruits/m^2^. Crop parameters such as stem elongation (cm per week), stem thickness (mm), fruit growth period (d), and truss formation rate (#truss/week) were manually measured per week on 10 sample plants. The stem thickness refers to the thickness of the stem just below the highest flowering cluster near the plant top. The fruit growth period refers to the time between the day that the first fruits on the cluster clearly start to grow and the day of harvest of the cluster. Plant load (#fruits/m^2^) was estimated from stem density, numbers of new and harvested trusses, and number of fruits per new truss, and was weekly shared with the teams. Harvest was performed per truss, approximately five times every two weeks. Harvest data on number of harvested trusses (#/m^2^) and fresh fruit weight (kg/m^2^) of class A, were obtained manually by the greenhouse staff. Additionally, fruit quality analyses were carried out in the laboratory. Based on laboratory measurements of total soluble solids (TSS, °Brix), titratable acid (Acid, mmol H_3_O+/100 g), % juice pressed from the fruit wall of the tomato (%Juice, %), breaking force of the fruit wall, as an indicator of the perceived firmness during chewing (Bite, N) and average fruit weight (Weight, g), the fruit flavor (0 = dislike, 100 = like) were calculated with the WUR Flavor Tomato Model version 2.1 (update 2011) [59]. Results were shared with teams every second week.

### 2.5. Resource Use Efficiency 

Resource use efficiency was calculated based on measured data: energy use efficiency for heat (MJ/kg tomato) and electricity (kWh/kg tomato), CO_2_ use efficiency (kg CO_2_ dosage/kg tomato), water use efficiency (L supplied/kg tomato), fertilizer use efficiency (g fertilizer/kg tomato). Fertilizer use efficiency was estimated based on the average supply EC and using the rough relation that 1 EC corresponds to 1 kg of dissolved salts per m^3^ of water.

### 2.6. Economics

**Figure A1:**
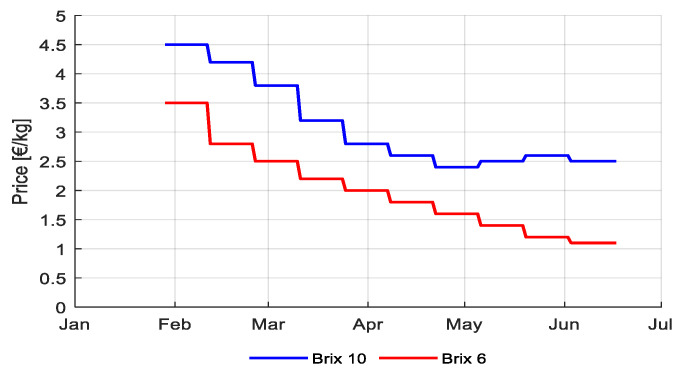
Tomato prices cv. *Axiany* (€/kg) depending on different Brix values (6 and 10) during the harvesting period (13 February until 29 May 2020).

Net profit was calculated based on income minus costs. The income was determined from the kg tomato fruits harvested x price per kg fruits and fruit quality. The price depended on fruit quality, namely its Brix value, and on the season (Figure A1). The costs were based on the operational costs related to resources used by the teams during the experiment. Initial costs for the young plants (costs of a young plant x number of young plants placed in the compartment) were € 2.00 for a 1-stem plant. and € 2.20 for a 2-stem plant. Resource use of electricity, heating, CO_2_, water, nutrients, and labor were measured during the experiment per greenhouse compartment and multiplied with the given price: electricity on-peak price (07:00–23:00 h) € 0.08 per kWh and off-peak price (23:00–7:00 h) € 0.04 per kWh; heating price € 0.03 per kWh; CO_2_ price € 0.08 per kg up to 12 kg/m^2^ and € 0.20 per kg above; labor for crop maintenance € 0.0085 per stem per m^2^ per day. Other greenhouse equipment used, was identical, and therefore capital costs were not considered in the calculation of the net profit. All economical parameters were communicated to the teams prior to the start of the challenge and had therefore no uncertainty.

### 2.7. Performance Analysis

Teams operated the different greenhouse compartments using their own AI algorithms. The outcome were different management strategies for climate, irrigation, and crop, affecting crop yields, product qualities and resource use efficiencies, and thus income, costs, and net profit.

In a performance analysis the realized results of the real greenhouse crop production in different compartments were compared with a greenhouse climate and crop simulation model, a virtual greenhouse crop production (digital twin). With the availability of this digital twin of the real greenhouse, a detailed analysis can be performed to better understand the roles of different growth factors such as light, temperature, CO_2_ etc.

The virtual greenhouse crop production model consisted of a combination of a dynamic greenhouse climate model KASPRO [24] and a tomato crop model INTKAM [43]. The combined model assumes adequate supply of water and nutrients and ignores the presence and effects of pests and diseases. The KASPRO model computes the greenhouse climate as a function of the realized outside weather conditions in our experiment and the realized greenhouse climate control settings in our greenhouse with the real parameters of construction and equipment. The model processes these control settings by a control algorithm comparable to the ones used in the real greenhouses. The climate model output consists of various climate parameters, such as light intensity, temperature, CO_2_ concentration and air humidity. This output is then used as input for the tomato crop model INTKAM [43], which computes daily gross photosynthesis from the sum of hourly photosynthesis rates. The hourly photosynthesis rates are the outcome of a dynamic crop architecture (leaf area index and plant load) under the dynamic climate conditions. Crop photosynthesis minus crop dissimilation results in the amount of carbohydrates produced. The daily amount of carbohydrates is then partitioned over the growing organs (roots, stems, leaves, fruits) according to their relative potential growth rates. Dry matter fraction and fresh organ weights are calculated in a next step. In a last step, the harvest moment of individual fruits is determined on its physiological fruit age [60], tomato yield results. The net profit is computed as described above.

The digital twin was used to calculate the tomato crop yield of each compartment, while using the real greenhouse construction, the real equipment, the real weather conditions and the realized climate and crop management strategies in the individual compartment as inputs. The calculated output was the predicted fresh yield (kg/m^2^). The crop model was appropriately calibrated. With this calibrated digital twin of the greenhouse, the effects of changes in light, air temperature and CO_2_ control strategies on fresh production were investigated for each compartment of the experiment. The influence of light availability was investigated by increasing or decreasing the number of lighting hours by max 3 h per day, not changing the applied light intensity. CO_2_ dosing capacity was varied from 50 to 200 kg/ha/h, while simultaneously changing the setpoint of CO_2_ concentration from −100 to +50 ppm compared to the applied strategy. Temperature setpoints were changed by −2 to 2 °C, compared to the applied strategy. Effects on net profit were investigated.

## 3. Results

### 3.1. Climate Strategies

**Figure 3:**
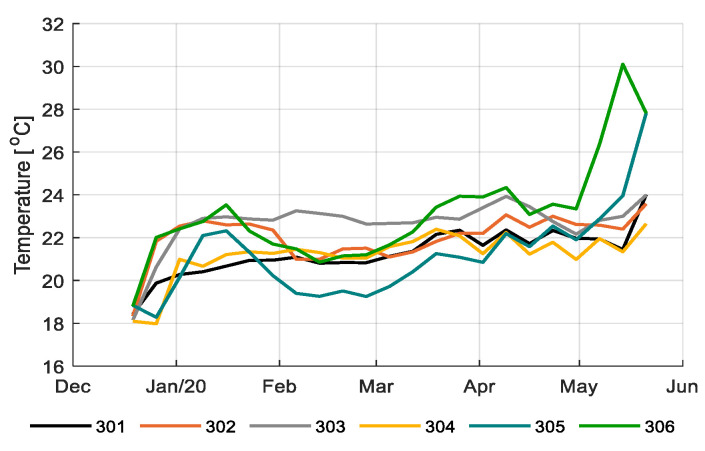
Weekly average temperature (°C) in the six greenhouse compartments (301–306).

**Figure 4:**
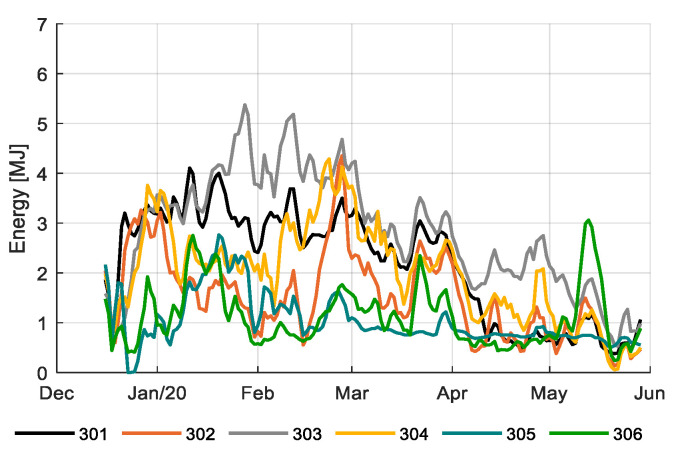
Heating energy usage (MJ) in the six greenhouse compartments (301–306). Data smoothed by a moving average filter of 3 days.

During the experiment, teams applied different climate strategies in their greenhouse compartments. Figure 3 shows the realized average temperature during the growing period for each compartment. Figure 4 shows the heating used. While some compartments showed a relatively stable temperature regime throughout the season (301, 303, and 304), others showed higher temperatures during the first weeks, then a moderate level and a large increase at the end (305 and 306), probably to accelerate development early in the season and fasten fruit ripening at the end of the season. While 305 and 306 appeared to apply the same concept, temperatures of 305 were lower than those of 306. Despite the high temperature regimes applied in 306 the team reached higher heat use efficiency (Table 2). This was achieved by allowing a high humidity and limit ventilation where possible (data not shown). The reference growers (303) applied relatively high temperatures throughout the season, which also resulted in the highest heating use (Figure 4; Table 2).

**Figure 5:**
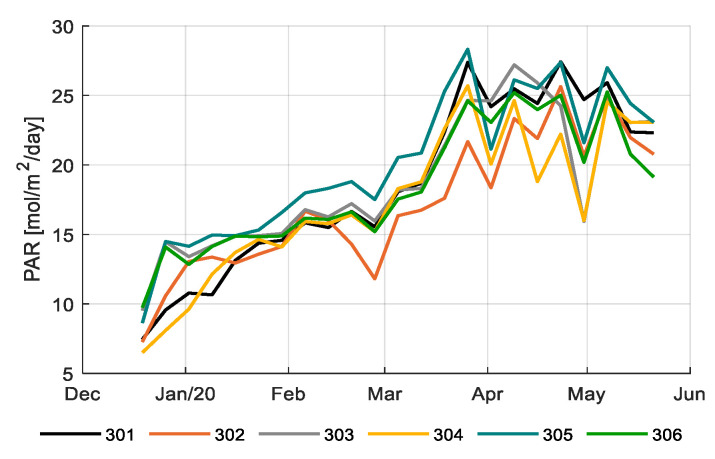
Weekly average total daily photosynthetically active radiation (PAR) light integral (mol/m^2^/d) (natural sunlight and artificial lighting) in the greenhouse compartments (301–306).

**Figure 6:**
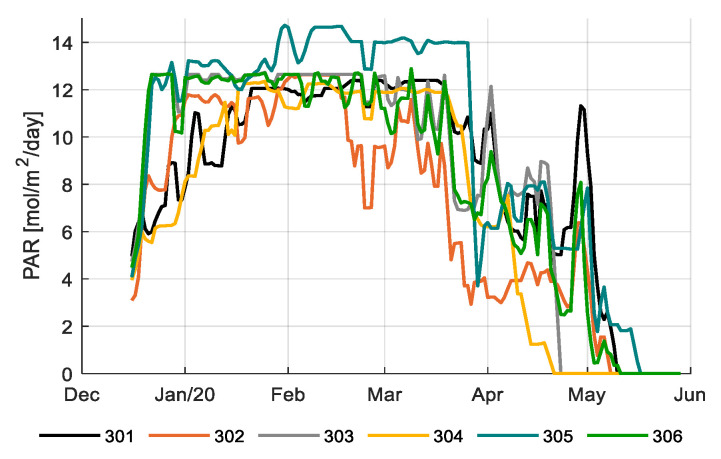
Daily PAR light integral (mol/m^2^/d) of artificial lighting only in different greenhouse compartments (301–306). Data smoothed by a moving average filter of 3 days.

Figure 5 shows the total daily integral of photosynthetically active radiation (PAR) during the growing period for different compartments. The daily PAR integral consists of the amount of natural light entering the greenhouse and the amount of artificial light (HPS and LED) added in the light control strategy. Figure 6 shows the daily PAR integral from artificial lighting only; 305 had the highest artificial lighting usage with 48% of the total PAR light came from the lamps; and 304 and 302 had lower artificial lighting usage with 41% of the total PAR light came from the lamps. Artificial lighting usage is reflected in electricity consumption (Table 2). The team with the best strategy (306) and the reference growers (303) had an average strategy, and thus electricity consumption.

**Figure 7:**
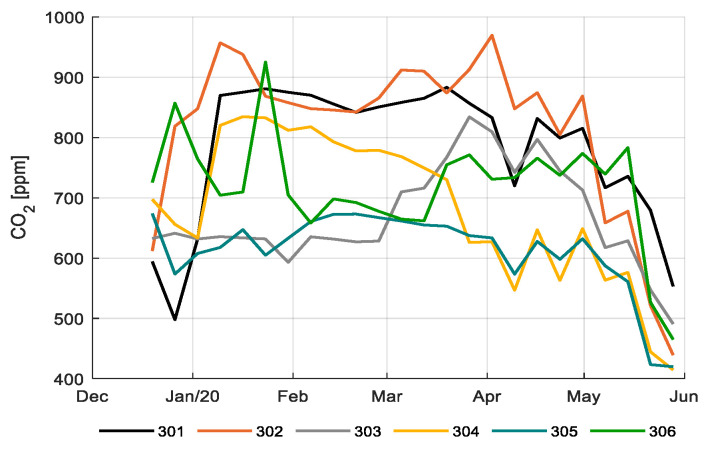
Weekly average CO_2_ concentration (ppm) during the light period in different greenhouse compartments (301–306).

**Figure 8:**
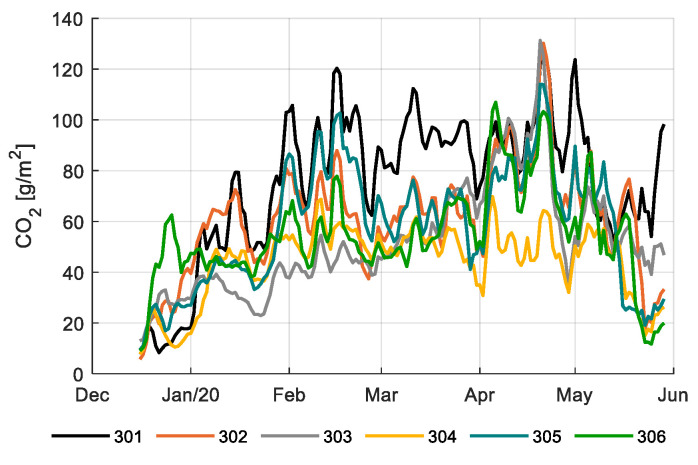
Daily CO_2_ dosage (g/m^2^) in different greenhouse compartments (301–306). Data smoothed by a moving average filter of 3 days.

Figure 7 shows the CO_2_ concentration inside the greenhouse compartments during the light period. Values vary between 600 and 900 ppm for most of the season, lowering to 400–600 ppm at the end of the season. Figure 8 shows the CO_2_ dosage. Compartment 301 and 302 maintained high CO_2_ levels throughout the season; 301 achieved that with very high CO_2_ dosage (Figure 8); 302 realized the same levels with lower dosage, both with comparable CO_2_ use efficiencies (Table 2); and 305 achieved much lower CO_2_ levels (Figure 7), however, resulting in comparable CO_2_ use efficiencies (Table 2) due to lower production (Figure 16). The reference growers (303) started with relatively low CO_2_ concentration and dosage but increased it at the end of the growing cycle (Figure 7 and Figure 8). Compartment 304 had the opposite strategy resulting in the best CO_2_ use efficiency (Table 2) since they maintained high CO_2_ levels during winter with low ventilation losses and lower CO_2_ levels during summer, which limits the ventilation losses.

### 3.2. Irrigation Strategies and Fruit Quality

**Figure 9:**
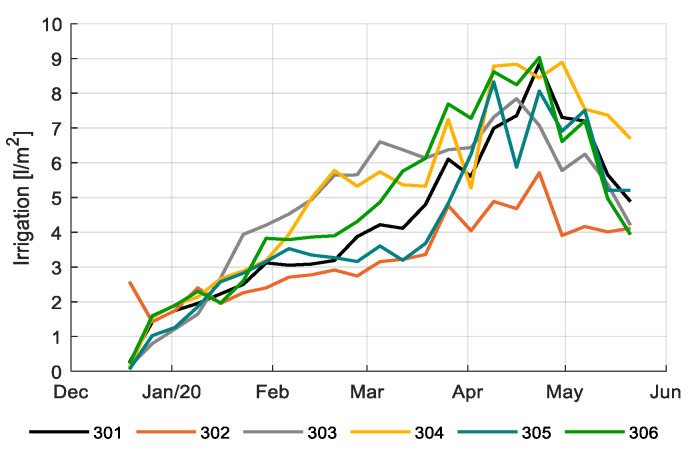
Weekly average of the daily amount of irrigation water (L/m^2^) provided in the different greenhouse compartments (301–306).

**Figure 10:**
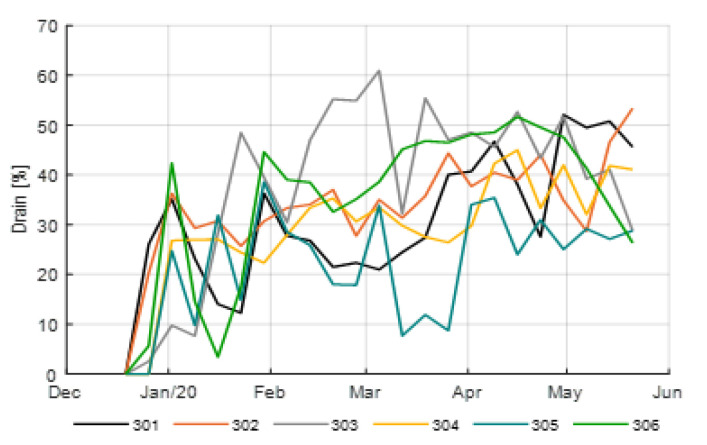
Weekly average of the drain percentages (%) applied in the different greenhouse compartments (301–306).

The amount of irrigation water provided in the different compartments differed substantially. This was caused by different irrigation supply strategies (Figure 9), resulting in different drainage (Figure 10), and by different artificial lighting and ventilation control (data not shown). Drain water was captured to be re-used, crop water uptake is less than the amount of water supplied. The amount of irrigation water supplied varied between 533 L/m^2^ for compartment 302 to 832 L/m^2^ for compartment 304. After subtracting the collected and re-used drain water, the crop water uptake was 334 L/m^2^ for compartment 302 and 537 L/m^2^ for compartment 304. The average usage was 450 L/m^2^, the team with the best strategy (306) used 430 L/m^2^.

**Figure 11:**
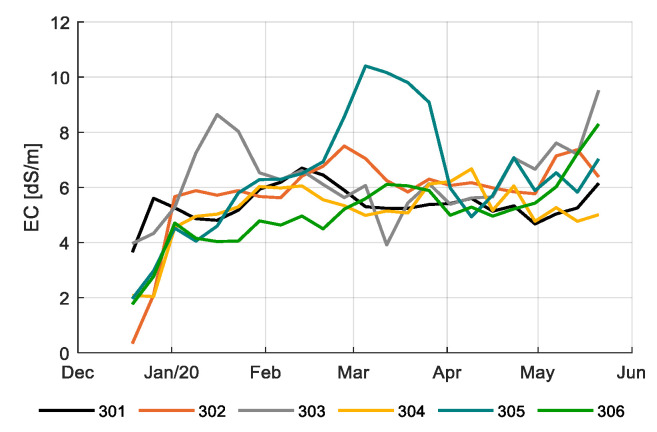
EC (dS/m) in drain water in the greenhouse compartments (301–306).

**Figure 12:**
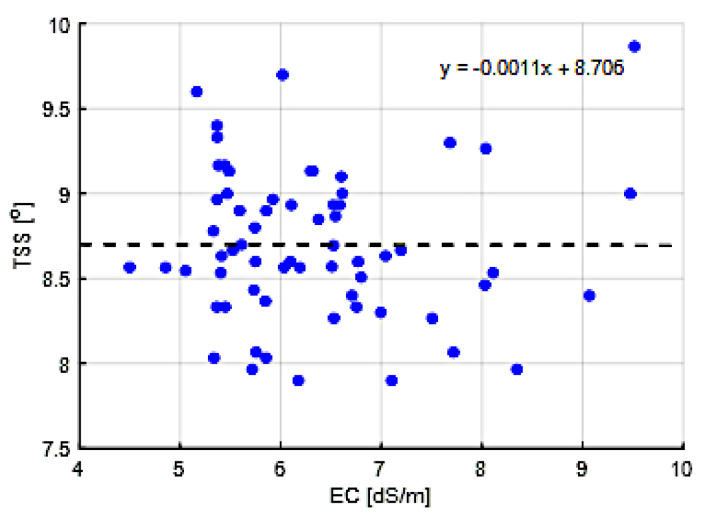
Correlation between total soluble solids (TSS, °Brix) and EC (dS/m) of the different greenhouse compartments (301–306).

**Figure 13:**
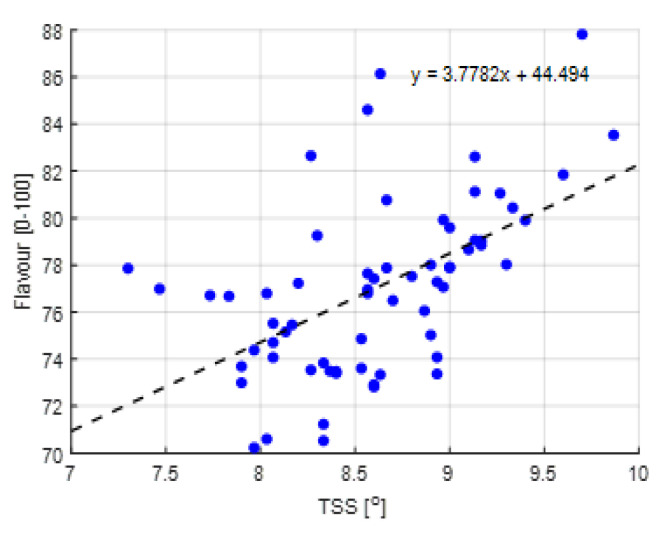
Correlation between TSS of tomato fruits (^o^) and fruit flavor (0 = dislike, 100 = like)) of the different greenhouse compartments (301–306).

Figure 11 shows the EC of the drain water from the different compartments during the growing season. The EC of the drain water is in general assumed to reflect the EC in the root zone. Teams were quite stable in EC, but a peak can be noted in compartment 305 in the middle of the growing period. Probably there was a short period where the control algorithm was not paying enough attention to the EC-control. In general, it is assumed that a high EC value induces high Brix and flavor ratings [61]. However, although compartment 305 showed a notably higher Brix value about four weeks after the high EC-values in the drain of compartment 305 (Figure 17), aggregated data in Figure 12 show that such a relation was not observed in the experiment. Still, the positive correlation between Brix and flavor was observed (Figure 13). Differences in Brix led to differences in prices, as shown in Figure A1.

### 3.3. Crop Strategies and Production

**Figure 14:**
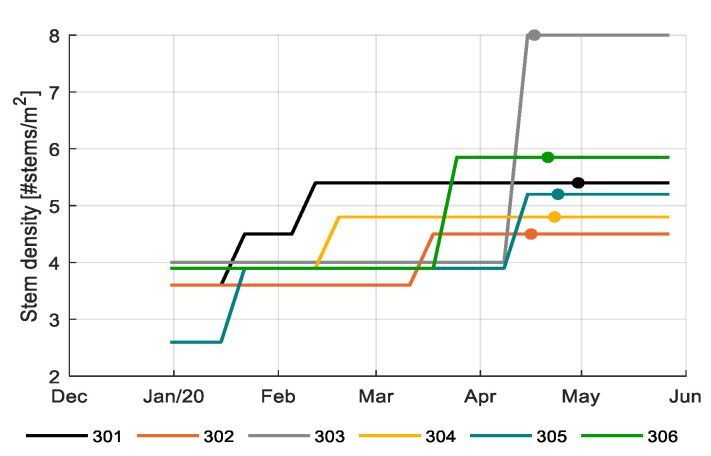
Stem density (# stems/m^2^) and topping dates (-o-) in different greenhouse compartments (301–306).

Figure 14 shows the initial stem densities of the different teams varying from 2.6 to 4.0 stems per m^2^. A low initial stem density reduced the costs for plant starting material. During the growing period stem densities were increased up to 4.5–5.8 stems per m^2^, by allowing shoots to develop to secondary stems. In the end phase of the crop cycle, just before topping, the reference growers (303) doubled stem density to 8.0 stems per m^2^. The purpose was to enable the development of two additional fruit clusters per stem. However, since labor costs were related to stems per m^2^, this action resulted in the highest labor cost (Figure A5), and a reduction in net profit (Figure A4), while boosting production at the end (Figure 16).

**Figure 15:**
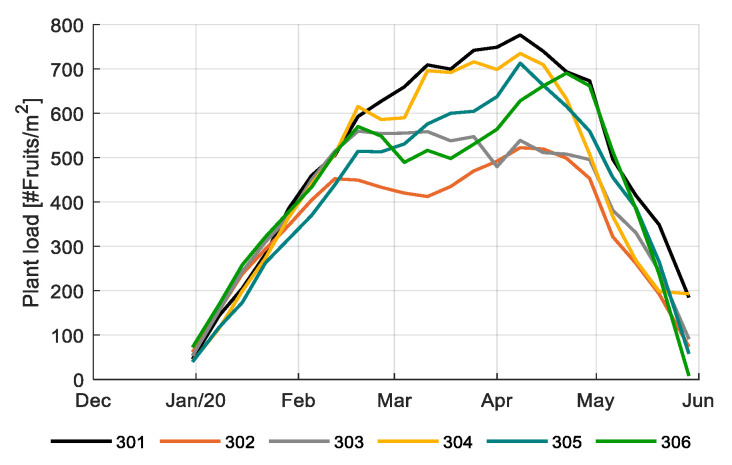
Plant load (fruits/m^2^) in different greenhouse compartments (301–306) realized by different teams during the experimental period (16 December 2019 until 29 May 2020).

Figure 14 and Table 1 show the chosen topping dates of different teams, varying from April 17 to 30. An early topping date ensures all remaining fruits to ripen until the end of the growing period; a too early topping date would leave no fruits to harvest towards the end (which was not the case in this experiment); a too late topping date would cause the crop to invest in new fruits without being able to ripen fully before the end (301 and 304, Figure 15). In spite of the variation in topping dates, plant load was zero at the end of the challenge, except for 301 and 304.

**Figure 16:**
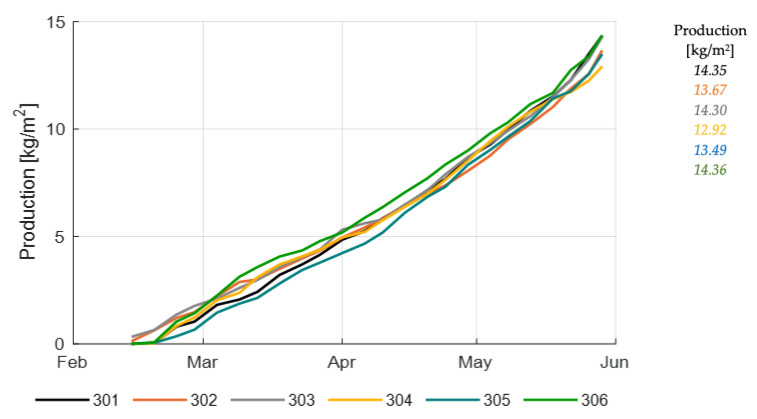
Cumulative and total production of class A tomato fruits (kg/m^2^) in different greenhouse compartments (301–306).

Table 1 shows relevant fruit development parameters. The final number of trusses per stem depends on the development rate, which is temperature dependent [36], and the period between planting and topping. The final number of trusses per stem varied between 21.7 (304) and 23.8 (301). The team with the best strategy 306 achieved an average number of trusses per stem (22.6). Number of trusses per stem, number of fruits per truss, and stem density together determine the total number of fruits per m^2^. Ultimately, 301 also had the highest number of fruits m^−2^ formed, whereas 302 had the lowest number of fruits m^−2^ formed. The team with the best strategy 306 had achieved the second highest number of fruits per m^2^ (Table 1), partly explained by the relatively high stem density of 5.85 stems m^−2^ from the end of March onwards (Figure 14). Number of fruits, together with the fruit weight, leads to total harvested fresh weight (Figure 16).

Figure 15 shows the plant load in different compartments during the experimental period. Plant load is determined by development rate, number of fruits per truss, number of fruits harvested (Table 1) and stem density (Figure 14) once the crop entered the generative state. It is associated with the dry matter partitioning towards fruits (sink of carbohydrates) from the leaves (source of carbohydrates), and therefore an appropriate parameter for monitoring source-sink balance and balance between vegetative (leaves) and generative (fruits) growth. As the plant load shows the number of concurrently growing fruits at each moment in time, its integral divided by the ripening time gives the final number of fruits harvested (Table 1). Compartment 301 and 304 maintained the highest plant load for most of the season (Figure 15). The lower plant load of 304 at the end resulted in a relatively decrease of production at the end (Figure 16). The most profitable strategy (306) maintained an average plant load with an increase towards the end; however, their plant load, fruit dry weight, fruit dry matter fraction, and all climate factors (Figure 3, Figure 5, and Figure 7) seemed to have been balanced enough to reach the highest total production throughout the season (Figure 16). A comparable plant load strategy was maintained by the reference growers, also leading to a high production (Figure 16), however due to also high resource use (Table 2) and labor costs net profit was lower (Figure A5).

Figure 16 shows the cumulative and total fresh production of tomato fruits class A in different compartments. Highest production was reached by the strategy in 306, by 301 and the reference growers (303). Only 306 was able to reach this high production also with a low resource use (Table 2); thus, a high net profit (Table 3). Production ranged from 12.9 to 14.4 kg/m^2^.

**Figure 17:**
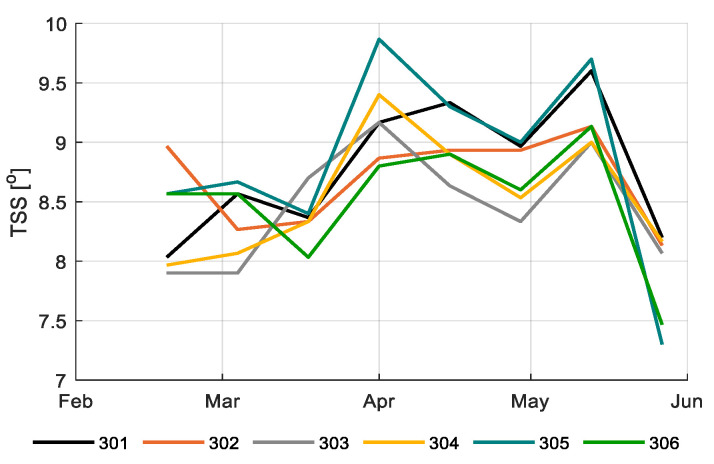
Brix of tomato fruits (^o^) in different greenhouse compartments (301–306).

**Figure 18:**
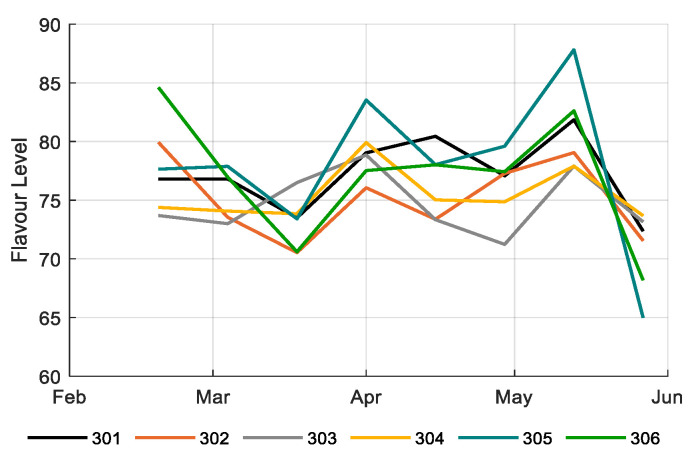
Flavor of tomato fruits (0=dislike, 100=like) based on calculations with WUR Flavor Tomato Model version 2.1 (2011), fruits from different greenhouse compartments (301–306).

**Figure A2:**
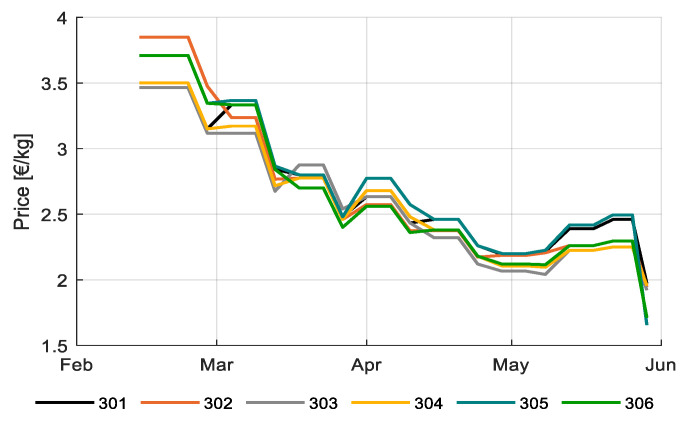
Realized tomato prices (€/kg) of different teams in different greenhouse compartments (301–306) during the harvesting period (13 February until 29 May 2020) based on their fruit quality (Brix).

Figure 17 and Figure 18 show tomato fruit quality over time in different compartments in terms of Brix and flavor, respectively; 305 reached highest Brix and flavor values, and thus highest prices (Figure A2), and the reference growers mostly reached low Brix and flavor values, thus relatively low prices (Figure A2). The best strategy 306 and the second best in ranking 302 reached average Brix, flavor and prices. However, both were able to have high quality at the beginning of the growing period, when prices were highest (Figure A1, Figure 17).

### 3.4. Resource Use Efficiency

Table 2 summarizes the resource use efficiency of all teams and compartments, a result of resources used for climate and irrigation control (Section 3.1 and Section 3.2) and realized crop production (Section 3.3). Reference growers (303) ranked lowest in resource use efficiency with a high usage of heating, water, and nutrients, but average usage of electricity and CO_2_; although, they realized a high production (Figure 16). Team 305 realized the best heating use efficiency and the lowest electricity use efficiency. Team 304 was opposite, which indicates that heating and lighting are partly interchangeable, as lamps do not only provide light, but additional heat loads. The team with the best strategy (306) managed not only to achieve the highest profit, but also a low consumption of resources. In heat, CO_2_ and fertilizer among the lowest and for electricity an average resource efficiency.

### 3.5. Economic Result

The net profit is for most of the growers the most important performance indicator. This means that one should aim for a high production and product prices, while minimizing resource use and costs associated. Table 3 shows and overview of realized total income, total costs, and net profit in different compartments.

**Figure A3:**
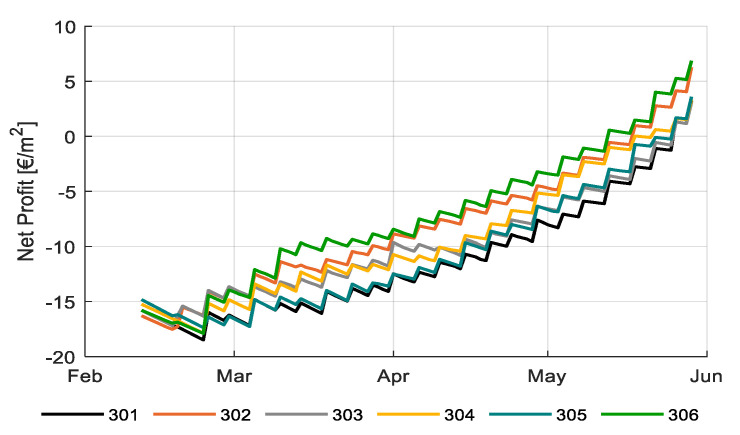
Cumulative income (€/m^2^) in different greenhouse compartments (301–306) realized by different teams during the harvesting period (13 February until 29 May 2020).

Total income consists of the amount of fruit harvest, fruit quality and product prices. Product prices per kg tomato were assumed to vary with fruit quality (Brix) during the growing period, reflecting market reality (Figure A1). Since fruit quality varied for different compartments during the growing season (Figure 17), product prices varied as well (Figure A2). Together with the realized fruit harvest (Figure 16) this was leading to the realized income (Figure A3, Table 3). Highest income was realized by the strategy in 306 (37.2 €/m^2^).

Total costs (Table 3) consists of the amount of resource use (Table 2) and costs of the resources (see Section 2.7). Lowest costs were realized by the second in ranking team, 302 (33.0 €/m^2^), while the best strategy has average costs (26.07 €/m^2^) and the reference growers were high in costs (29.38 €/m^2^), especially very in high energy and labor costs (Figure A5).

**Figure A4:**
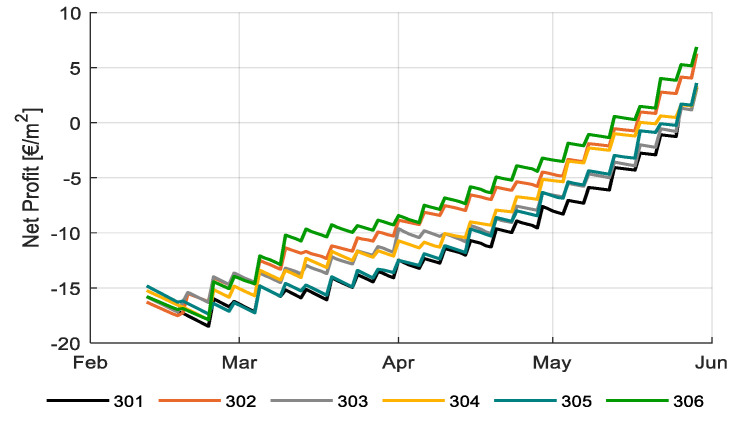
Net profit development (€/m^2^) realized by different teams in different greenhouse compartments (301–306) during the harvesting period (13 February until 29 May 2020) and total net profit (€/m^2^).

**Figure A5:**
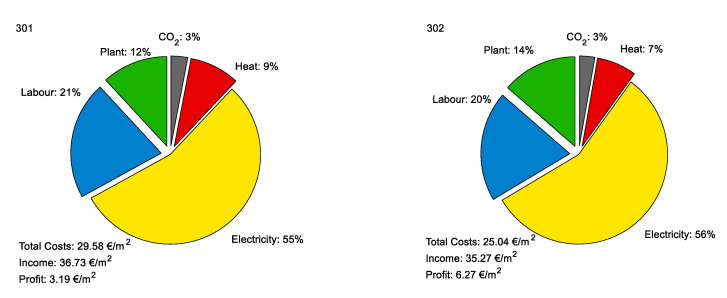

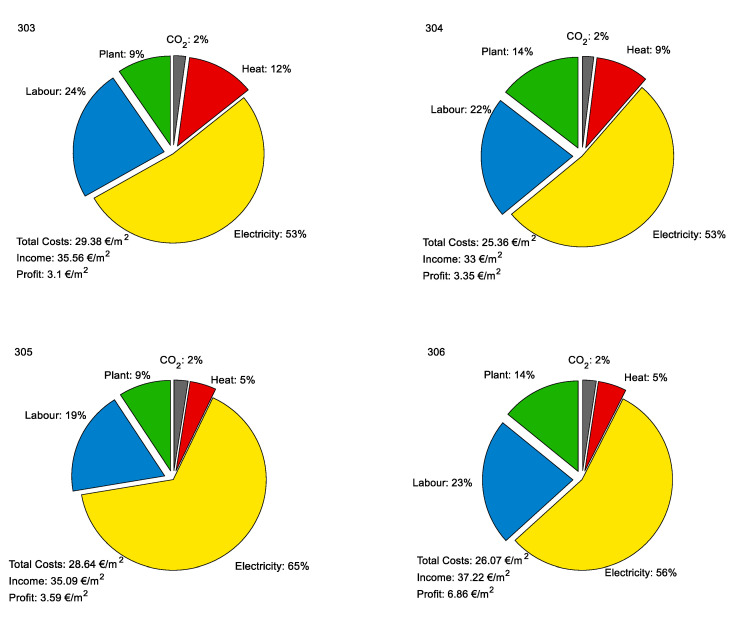
Net profit (€/m^2^), different realized cost components (heat, electricity, CO_2_, plant and labor), income and net profit in different greenhouse compartments (301–306) during the experiment.

The ranking in net profit is shown in Table 3. The team with the best strategy realized highest net profit with highest income and average costs, while the reference growers realized the lowest in net profit with average income, but high costs. The net profit development is shown in Figure A4, income development in Figure A3, while more detailed information on cost components is given in Figure A5. Net profit ranged from 3.10 to 6.86 €/m^2^. All AI operated greenhouses were able to reach higher net profits than the reference.

### 3.6. Performance Analysis

**Figure A6:**
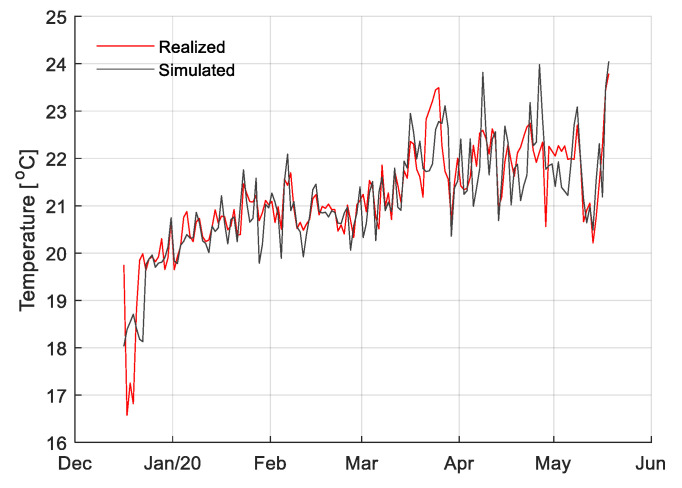
Measured and simulated greenhouse air temperature (°C) after automated distillation of climate strategies during the experimental period (16 December 2019 until 29 May 2020) for one of the compartments (301).

**Figure A7:**
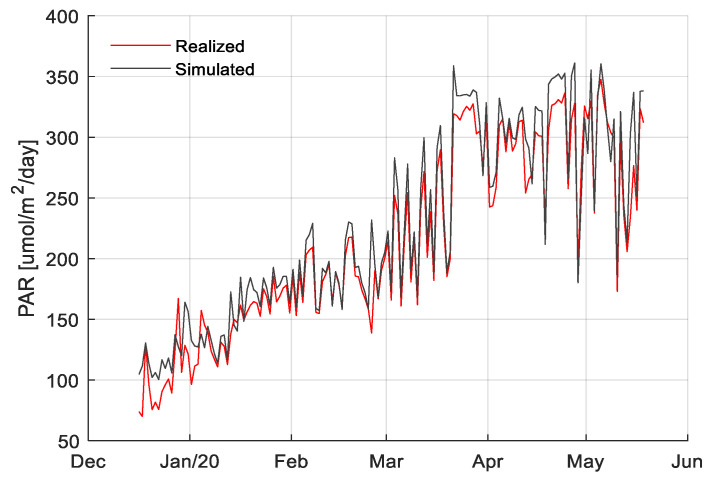
Measured and simulated average PAR (μmol/m^2^/d) after automated distillation of climate strategies during the experimental period (16 December 2019 until 29 May 2020) for one of the compartments (301).

**Figure A8:**
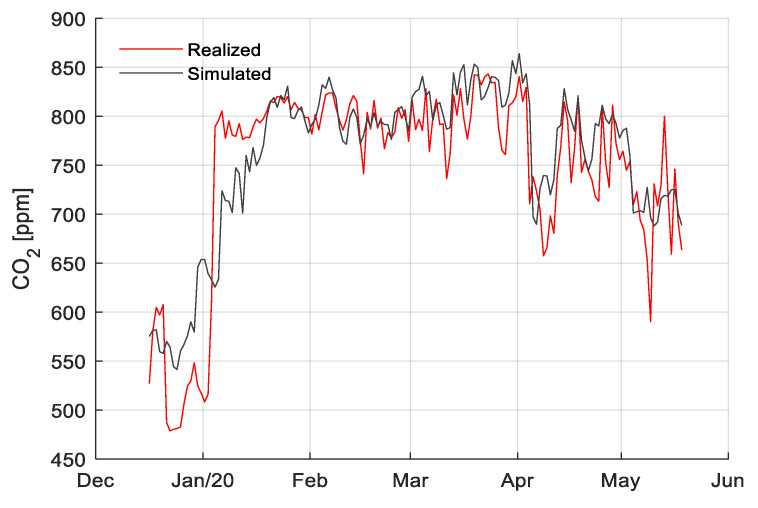
Measured and simulated CO_2_ concentration (ppm) of the greenhouse air after automated distillation of climate strategies during the experimental period (16 December 2019 until 29 May 2020) for one of the compartments (301).

**Figure A9:**
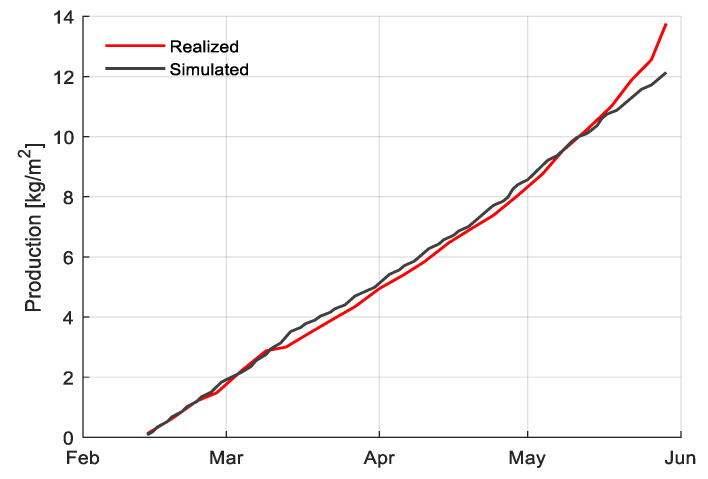
Measured and simulated cumulative fresh weight production (kg/m^2^) of the after calibration of the models with the actual climate and crop strategies during the experimental period (16 December 2019 until 29 May 2020).

The availability of the well validated greenhouse climate and crop models KASPRO-INTKAM; thus, the availability of a digital twin of the greenhouse tomato production, allowed the use of the models for a quantitative performance analysis. After automated distillation of important climate strategies (temperature, CO_2_, and lighting) from the existing realized data, simulation results of important climate parameters, such as temperature (Figure A6), CO_2_ (Figure A7) and light (Figure A8) were compared with realized climate parameters. Data shows that simulated and realized results are comparable. After appropriate calibration of the crop model, simulated fresh weight of harvest (Figure A9) was compared to realized crop production. Data shows that the simulated and realized results are comparable during the largest part of the harvest period. However, Figure A9 shows that the final boost in the last two weeks after topping was not captured. Further improvements on the model would be needed for a closer match in these last weeks by refinements on the dry matter partitioning between generative and vegetative parts and the gradual increase of the ripening speed after removal of the plant apex. A more in-depth analysis of the performance during this period was not conducted.

After this model validation, a performance analysis was carried out, analyzing the effects of changes in control strategies on fresh production and net profit for each compartment of the experiment. As crop growth is predominantly affected by the availability of light and CO_2_ and the greenhouse has to be at a favorable temperature to allow a proper growth and development of the crop, a sensitivity analysis was carried out on changes in these three major parameters. Data shown on net profit only.

**Figure 19:**
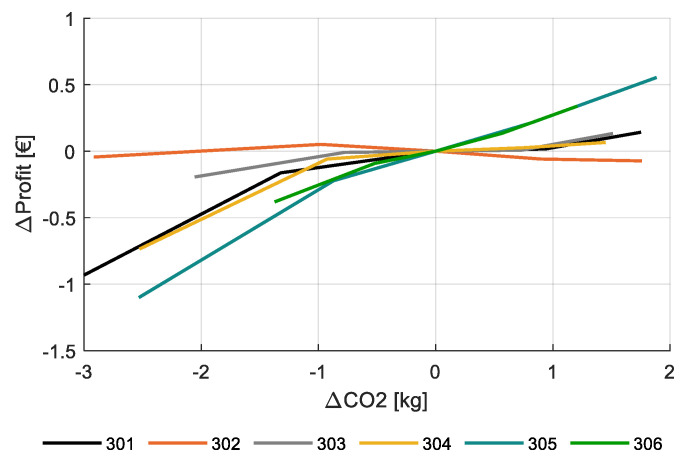
Change in simulated net profit (€/m^2^) for varying CO_2_ supply (kg/m^2^) for the different greenhouse compartments (301–306).

Figure 19 shows the simulated effect of variations in CO_2_-supply on net profit with respect to the strategy as applied by the different teams. Each team has controlled the CO_2_-conentration in a different way resulting in a range of CO_2_ dosing from 7.2 kg/m^2^ for compartment 303 to 11.7 kg/m^2^ for compartment 301. The sensitivity was evaluated by changing the CO_2_ setpoint by −100 to +50 ppm, while also changing the dosing capacity from 50 to 200 kg/ha/h. This resulted in a changed CO_2_ supply (kg). The lowest CO_2_ setpoints, reduced the production for some teams substantially, up to 0.72 kg/m^2^, representing a value of €1.80 per m^2^ for team 301. At a CO_2_ price of €0.08 per kg it is clear that the reduction of income is far more than the savings on CO_2_, so reduction of CO_2_-dosing is not beneficial for profitability. When increasing CO_2_ dosing, there is some benefit to gain, but the benefit is small for most teams, which show that the teams were operating at quite an optimal strategy.

**Figure 20:**
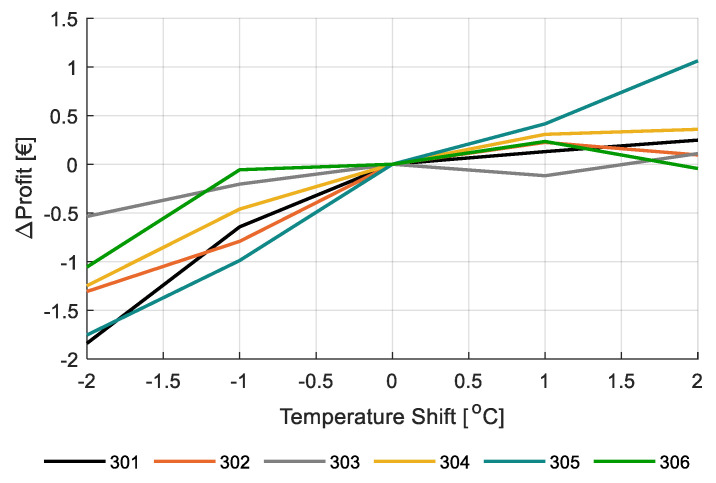
Change in simulated net profit (€/m^2^) for varying temperature shifts −2 to +2 °C on the applied heating temperature strategy for the different greenhouse compartments (301–306).

Figure 20 shows the effect of variations in temperature on net profit with respect to the strategy as applied by the different teams. Temperature was varied by −2 to 2 °C, compared to the applied strategy. This analysis shows that a decrease may save some on heating, but the lowered production leads to a decreased net profit. Increase of the temperature was simulated to be profitable although the increment in profit was again small. It can therefore be concluded that according to the simulation model, with changes in temperature potential gains were small.

**Figure 21:**
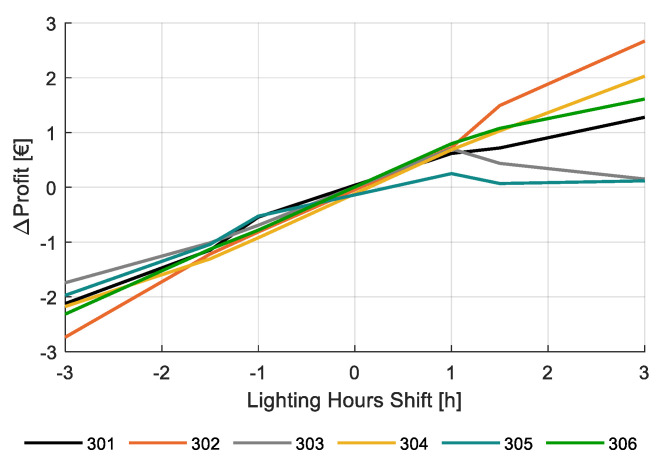
Change in simulated net profit (€/m2) for varying the daily lighting hours −3 to +3 h on top of the realized illumination strategy for the different greenhouse compartments (301–306).

Finally, Figure 21 shows the effect of variations in artificial lighting on net profit with respect to the strategy as applied by the different teams. The influence of light availability was investigated by increasing or decreasing the number of lighting hours by max 3 h per day, not changing the applied light intensity. An increase of lighting will result in higher electricity costs, higher production, and a little less cost for heating. A decrease of the number of lighting hours will generally do the opposite. It can be observed that the response of net profit on the application of light is much stronger than the response of changes in CO_2_ dosing and temperature. Moreover, the response seems to be quite linear, except for team 303 and team 305, which already applied a lot of lighting in the reality.

**Figure 22:**
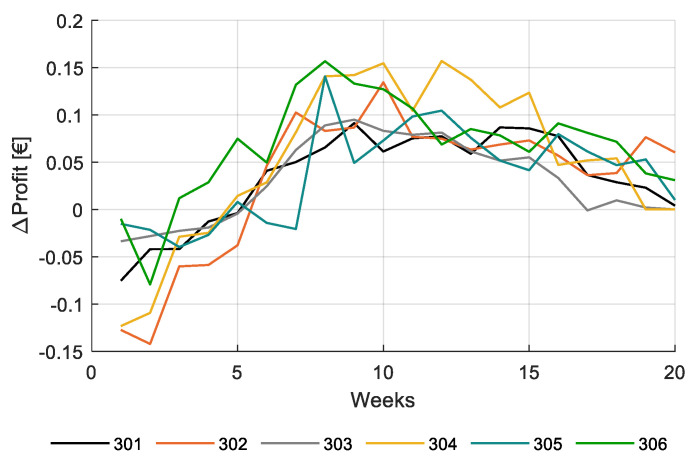
Change in simulated net profit (€/m^2^) for additional +2 illumination hours for each individual week while maintaining the remaining illumination strategy unchanged for the different greenhouse compartments (301–306).

The strong effect of light on net profit in the performance analysis requires a somewhat deeper analysis. Because of the large increase of the amount of natural sunlight towards summer (Figure 5) and the reduction of product prices towards summer (Figure A1), the revenue of additional artificial light can be expected to differ during the growing season (16 December 2019 to 29 May 2020). To analyze the effect of artificial light in time, the effect of two additional hours of artificial lighting per day, for each week during the growing season, while maintaining the illumination for the other weeks un-changed. The result on net profit throughout the growing season is shown in Figure 22.

Figure 22 shows that the additional 2 h of artificial lighting during 1 week, thus 14 additional hours per week, resulted in an increase of net profit of around 0.1 €/m^2^ when applied around the tenth week after planting (planting 16 December 2019, thus in February and March) and in a strong drop towards the end of the growing period. The small effect at the end of the growing period can be expected, based on the increase of natural light and the decrease in product prices. However, the simulation also shows that in the first weeks after planting, adding additional artificial lighting leads to a decrease in net profit. The crop then is still small and only few fruits act as a sink for carbohydrates. Control of artificial light could be based on crop source-sink balance throughout the season, of which plant load (Figure 15) could be a measure. The data in Figure 22 shows possibilities of intelligent control on crop-based parameters. 

**Figure 23:**
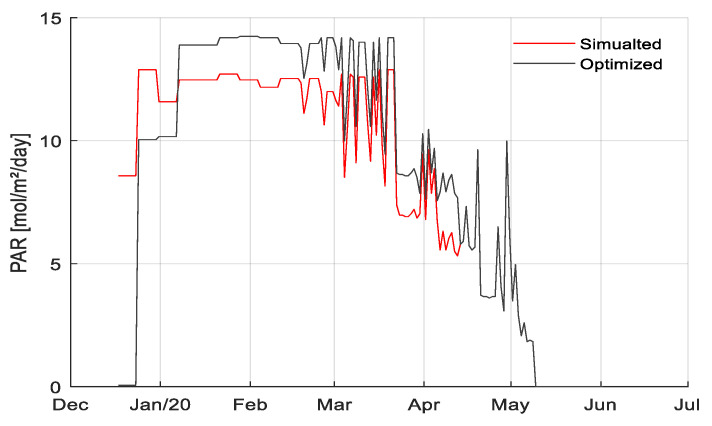
Daily amount of light (mol/m^2^/d) as applied in reality (blue line) and the simulated optimized amount of light (red line) leading to optimized net profit in compartment 306.

To explore the potential of an optimized lighting strategy, an algorithm was implemented that changed the amount of lighting hours per week per compartment (301–306) until the computed net profit showed a maximum for the particular compartment. An increase in net profit of 3.6 €/m^2^ for team 302 was simulated, which was the team with the lowest application of artificial light. An increase in net profit of 1.4 €/m^2^ was simulated for the team with the best strategy 306 with the highest realized net profit, if they would have applied a little less artificial light in the beginning of the growing season and somewhat more at the end. Figure 23 shows the daily amount of light as applied in reality in the lighting strategy of the team with the best strategy 306 versus the simulated optimized amount of light leading to optimized net profit. For simplification reasons, interactions with other growth factors, such as temperature or CO_2_, were not investigated. The methodology for performance analysis shown here could be applied for analyzing more details in the future.

## 4. Discussion

### 4.1. Cropping Strategy

Profitable tomato production implies the production of the maximum number of fruits of a certain quality while using a rational amount of resources. The product quality is determined by the size (too small fruits have a lower price) and Brix (fruits with higher Brix have a higher price, depending on season). With the choices in cultivation, growers aim on realizing a certain fruit size. As fruit size is negatively correlated to the number of fruits (per stem, per m^2^), reaching the target fruit size means that the grower has to manage the number of fruits produced. The number of fruits is determined by the number of trusses per stem, the number of fruits per truss and the stem density. In order to obtain the desired total number of fruits, the number of fruits per stem is to a certain extent interchangeable with stem density. However, given the fact that in this challenge the fruits were harvested and sold per cluster, the maximum number of fruits per cluster is constrained. This was done in order to limit the difference in maturity of the consecutive fruits within a cluster [60]. It limits the number of fruits on a truss to maximal 16 for single, and 20 for split trusses. Realized data on crop performance (Figure 14, Figure 15 and Figure 16; Table 1) show that some teams opted for a large number of fruits and a lower number of stems per m^2^, which saved on labor, where other teams applied a higher stem density and a lower number of fruits per cluster, which promoted evenly ripened clusters. It could be concluded that these are important parameters for optimization. Automated recording of relevant crop data is needed in the future to allow automated optimization.

It was assumed that fertigation would have an effect on fruit quality, and therewith on profitability. Whereas it is often assumed that the EC in the root zone has a positive effect on the Brix value of the tomato fruits [61], for this cherry tomato variety a good correlation could not be found. Figure 12 shows the correlation of measured Brix and average EC in the drain value during the 35 days prior to the harvest, which, for ease of computation, was considered as the fruit growth period. Combining all data, there was no effect of the EC in the drain on the Brix, which was on average 8.7. Similarly, there was no effect of the EC in the drain on the fruit dry matter content, which was in average 9.0% (data not shown). A parallel peak in EC and Brix values was observed halfway the harvest period for 305 but this was not a trend as other compartments also showed periods with a notably higher EC that did not result in an increased Brix. It could be concluded that the data and available knowledge on the used cherry tomato variety do not allow for automated optimization yet.

In the performance analysis, simulations with the virtual greenhouse, and crop model were carried out to analyze the effect of different growth factors on crop production and net profit. Cumulative fresh production of cherry tomato could be well parameterized and simulated, apart from the under-estimated production at the end of the growing cycle that was observed for all compartments. The dry matter partitioning in the INTKAM model is based on the potential growth rates of organs, and apparently, these rates are not adequately described for a tomato plant that has been topped.

Simulations with the virtual greenhouse and crop model demonstrated that net profit of the crop production was not very sensitive to CO_2_ dosing strategy. However, when the CO_2_ dosing was strongly reduced a clear reduction in production and net profit could be observed. In general, increasing CO_2_ dosing compared to the levels already applied by the teams hardly increased net profit. High levels of CO_2_ dosage during summer did not result in high air CO_2_ concentration, and therefore not in increased production, because window opening led to loss of the supplemented CO_2_. In winter, when windows are closed, CO_2_ dosage does in general lead to elevated air CO_2_ concentration and production increase. The limitations then are set by the crop that knows limits to the amount of CO_2_ it can absorb and convert into carbohydrates. However, teams seem to have used high enough CO_2_ concentrations (Figure 19). It could be concluded that at increased dosing capacities and CO_2_ setpoints, the additional revenue of the crop hardly exceeded the additional costs for CO_2_ beyond the realized CO_2_ strategies applied in the experiment. However, the control of CO_2_ clearly offers possibilities for autonomous control to optimize CO_2_ dosage in relation to ventilation management (costs) versus CO_2_ concentration and crop production (income).

Simulations with the virtual greenhouse and crop model were also carried out to study the effect of temperature on crop production and net profit. The effect of temperature on crop growth and development has a number of aspects and is therefore complex. With regards to photosynthesis, the effect temperature is small within moderate ranges (18–24 °C) [62], which implies that gross carbohydrate production will not change much. Temperature has a much stronger effect on maintenance respiration [63], so, increased temperature leads to reduced carbohydrate availability for growth. There is also an effect on the number of trusses developed. This is assumed to be linear [36], which was confirmed in the current experiment. Within the temperature ranges applied in this project, the truss formation rate varied from 1.1 cluster per week at 19°C diurnal average temperature to 1.47 cluster per week at 24 °C diurnal average temperature (data not shown). If other growth factors stay the same, this would lead to more, but smaller fruits. Since smaller fruits might reduce the product price, a good grower balances temperature (=number of trusses formed), number of fruits per truss and number of stems per m^2^ in such a way that the maximum number of fruits with a satisfying fruit weight is produced. Stem density is a strategic decision that can be changed only sporadically during the growing season, number of fruits per truss can be modified weekly, and temperature can be controlled at any moment; although, the response of the tomato crop in terms of production is based on the temperature management over at least several days or one week. It could be concluded that the temperature strategy offers clear opportunities for autonomous control. Since temperature management influences the number of trusses formed through truss formation rate and fruit ripening time over time, camera systems could automatically detect these parameters. The other two parameters, number of fruits per truss and the number of stems per m^2^, can be optimized by, e.g., sensors or model estimates of the photosynthetic capacity of the crop.

Simulations with the virtual greenhouse and crop model were also carried out to study the effect of lighting strategies on crop production and net profit. There is a strong difference between the instantaneous effect and the integrated daily effect of light on crop photosynthesis. As long as light is the limiting factor, its increase will cause a higher photosynthesis rate. However, even if instantaneous photosynthesis has reached on optimum, the integrated daily value will change if the light period is lengthened. This is the reason why more light caused more simulated growth and production (Figure 21). However, the response is not the same during the entire growing season. It is assumed that juvenile plants are sink limited in their growth, meaning that there are more carbohydrates produced than the small plants can process, which has also been incorporated in the crop simulation model. Increase of light in this phase only adds to costs, not to extra growth. There follows a transition phase during which growth changes from sink to source limited growth. An adult crop knows a source limited growth, and extra artificial lighting during this phase was proved to be financially profitable. However, as the amount of solar light increase and the value of the crop decreases towards the end of the growing period, the profitability of extra artificial lighting drops. It was shown that an algorithm optimizing the application of artificial light on its economic viability encourages artificial lighting in winter and early spring but discourages the use in summer. It could be concluded that the lighting strategy offers clear opportunities for automated optimization on source-sink balance of the crop.

### 4.2. Sensors, Algorithms, and Control

Greenhouses are highly non-linear, complex, multi-input and multi-output (MIMO) systems [64]. The underlying production processes present differences in response times of the variables involved. Greenhouse climate and crop photosynthesis respond rapidly to changes in control and external inputs, whereas crop growth and production respond comparatively slow to changes in control [17]. Whereas greenhouse climate information is available with many datapoints, crop related information is sparsely available. However, the performance of machine learning algorithms (e.g. deep learning, neural networks), highly depends on the diversity and size of training data [65].

Prior to the start of the greenhouse crop experiment, teams could explore, build, and train algorithms using a virtual greenhouse environment emulated by the available climate-crop models [24,43]. The use of synthetic training datasets has been shown to be very useful in earlier applications, when real-world data are not quantitatively and qualitatively sufficient for training purposes [51,66]. In the real growing experiment, contextually relevant data was collected via standard sensors, next to that data was collected by teams with additional sensors of their preference (see Section 2.3) to improve and increase the efficiency and robustness of their algorithms. During this exploration phase prior to the start of the experiment, teams should have accounted for such a systems’ architecture that would not be hampered by the data availability during the transition from the virtual to actual growing environment. In other words, teams that selected model-based control using the available models, should have trained their algorithms with parameters/data that they would be able to monitor in the greenhouse experiment.

Additional sensors of the teams varied from low-cost tailor made, to novel sensors for climate and crop monitoring. Natural and mechanical ventilated climate sensors for temperature, humidity, and CO_2_ as well as NIR and PAR sensors were placed by teams on different heights, to monitor climate homogeneity. Spatial mapping of the parameters supported algorithms of the teams on climate control decisions (ventilation, CO_2_ dosage, lighting strategy) and crop management (stem density, fruit pruning). The digital images of optical low- and high- resolution camera systems RGB, and thermal cameras allowed teams to remotely and visually inspect crop growth and well-being, monitor the number and ripening of the fruits as well as the temperature of the leaves at different crop heights. Wide-view cameras enabled the control of operations of actuators (e.g. lamps on/off, screen opening). Some teams labelled the collected imagery datasets based on expert knowledge and used them for phenotyping certain crop traits. In literature, several authors focused on spatial mapping of climate or digital image data for greenhouse growing crops [67,68,69,70,71,72]. Here are still many opportunities for automated computer vision analysis and automated control in the future. Mechanical sensors collected direct feedback from sampled plants. Weighing gutters and crop load cells allowed real-time monitoring of the crop and plant weight. Such data were potentially used by the teams in finding correlations between crop and plant weight changes under different climate, irrigation and crop management decisions that could support their algorithms. Furthermore sap-flow and stem diameter sensors on fixed positions allowed automated monitoring of fluid transport and stem diameter. The information was used by some teams for defining their heating temperature and irrigation strategies as previous research [73] reported associations of leaf temperature, stem and sap flow measurements with the water status of the plant and drought stress.

Each team followed a different approach for determining their strategies and controls of the greenhouse climate and crop. However, the majority decided to breakdown the system into long- and short-term decisions and controls. The decision-making scheme of team 306 consisted of three managerial levels: strategic, tactical, and operational. The strategic level aimed at defining their crop strategy (e.g., decisions on number of stems, leaf pruning, and temperature-light ratio). It received historical data, expert knowledge, digital images and crop registration data as input. Tactical decisions defined their climate strategy and generated 24-h baseline setpoints based on weather forecast, outputs of the strategic level, and indoor climate measurements. The operational level received data on the short term anticipated outdoor conditions, 24-h setpoints from tactical level and climate measurements, to generate climate control setpoints. To find an optimal greenhouse climate control the team explored model predictive control (MPC) and non-linear model predictive control (NMPC) framework to address the non-linear dynamics. Computation of their optimal control policies was conducted using data-enabled predictive control (DeePC). Using weather forecast and historic data and real time feedback their single optimization framework determined a non-parametric model representing the dynamics of the system, estimated the states and optimized the systems trajectories for a defined horizon [74]. For irrigation, the team applied an irrigation control algorithm that related total solar radiation and the gradient water content (WC) in the slab after the last irrigation [74]. Control of temperature and humidity through ventilation was managed with direct control of the windows position instead of relying on ventilation temperature and P-band usually applied by the climate computer. Finally, historical data, crop parameters coupled with weather forecast were used to classify stomata behavior and optimize window opening.

Other teams explored the use of long-short-term memory (LSTM) or bidirectional LSTM (BiLSTM) or tried reinforcement learning algorithms. AI algorithms were combined with conditional (rule based) decisions and expert policies based on historical recorded or empirical data for either climate or crop growing strategies or both. We can conclude that none of the teams had implemented a fully autonomous AI based control yet. All teams used humans in the decision loop. Further improvements towards fully autonomous control still have to be made in the future.

## 5. Conclusions

- In the experiment described here all teams remotely controlling the greenhouse tomato crop production by AI outperformed the human reference growers.
- Crop management has been shown to be important for high (quality) production.
- Optimizing lighting strategies would have improved the production and net profit of the team with the best strategy more than optimizing CO_2_ or temperature.
- There are clear opportunities for autonomously control crop growth based on automated control of lighting, CO_2_, temperature, and source-sink balance of the crop.
- Objective is data needed on all aspects of growing since the lack of data hampers further development of AI and/or optimum control strategies.
- Objective data can be obtained by specific crop sensors, especially the further development of robust camera’s and computer vision algorithms to detect crop specific parameters (e.g., plant load) seem to be interesting to improve in the future for fully autonomous growing.
- The last step towards fully autonomous growing would be to automate also all crop handling, more development on robotics would be needed for that (not part of this research).

## Figures and Tables

**Table 1:** Final number of trusses (#/stem) and number of fruits (#/stem, #/m^2^), average air temperature and topping dates in different greenhouse compartments (301–306), planting date 16 December 2019.

Greenhouse Compartment	Number of Trusses (#/stem)	Average Temperature (°C)	Number of Fruits (#/stem)	Number of Fruits (#/m^2^)	Topping Dates
301	23.8	21.34	332	1577	30 April 2020
302	22.0	22.04	292	1165	16 April 2020
303	23.2	22.70	302	1323	17 April 2020
304	21.7	21.37	325	1373	23 April 2020
305	22.0	21.40	351	1340	24 April 2020
306	22.6	23.25	315	1459	21 April 2020

**Table 2:** Resource use efficiency (unit resource used per kg tomato produced) for different teams and their crop in different greenhouse compartments during the experimental period, heat (MJ/kg), electricity (kWh/kg), CO_2_ (kg/kg), water (L/kg), and nutrients (g/kg).

Greenhouse Compartment	Heat (MJ/kg)	Electricity (kWh/kg)	CO_2_ (kg/kg)	Water (L/kg)	Nutrients (g/kg)
306	12.9	18.7	0.63	25.0	83.0
302	18.5	17.6	0.74	25.2	81.0
301	25.3	19.9	0.87	25.9	78.0
304	25.9	17.7	0.56	26.9	90.0
305	12.8	24.0	0.72	27.9	100.0
303	33.0	19.0	0.60	27.4	99.0

**Table 3:** Total costs, total income, and net profit (€/m^2^) for different teams and greenhouse compartments (301–306) during the experimental period (16 December 2019 until 29 May 2020).

Greenhouse Compartment	Total Costs (€/m^2^)	Total Income (€/m^2^)	Net Profit (€/m^2^)
306	26.07	37.22	6.86
302	25.04	35.27	6.27
305	28.64	35.09	3.59
304	25.36	33.00	3.35
301	29.58	36.73	3.19
303	29.38	35.56	3.10

## Supplementary Materials

The complete dataset of the second Autonomous Greenhouse Challenge and basis of this analysis is published online at https://doi.org/10.4121/uuid:88d22c60-21b3-4ea8-90db-20249a5be2a7.
